# *Dilochiadeleoniae* (Orchidaceae), a new species from Mindanao, Philippines

**DOI:** 10.3897/phytokeys.139.46935

**Published:** 2020-02-04

**Authors:** Danilo N. Tandang, John Michael M. Galindon, Edwin R. Tadiosa, Fulgent P. Coritico, Victor B. Amoroso, Noel E. Lagunday, Rene Alfred Anton Bustamante, Darin S. Penneys, Peter W. Fritsch

**Affiliations:** 1 Botany and National Herbarium Division, National Museum of the Philippines, Padre Burgos Drive, 1000 Ermita, Manila, Philippines National Museum of the Philippines Manila Philippines; 2 Center for Biodiversity Research and Extension in Mindanao (CEBREM), Central Mindanao University, Musuan, Bukidnon, Philippines Central Mindanao University Musuan Philippines; 3 Philippine Taxonomic Initiative, El Nido, Palawan, Philippines Philippine Taxonomic Initiative El Nido Philippines; 4 University of North Carolina-Wilmington, Wilmington, NC 28403, USA University of North Carolina-Wilmington Wilmington United States of America; 5 Botanical Research Institute of Texas, 1700 University Drive, Fort Worth, Texas 76107-3400, USA Botanical Research Institute of Texas Fort Worth United States of America

**Keywords:** biodiversity, Mount Hamiguitan Range Wildlife Sanctuary, orchid taxonomy, world heritage site

## Abstract

A new species, *Dilochiadeleoniae* Tandang & Galindon (Orchidaceae), from Mindanao Island, Philippines is described and illustrated herein. This species is distinct from other known Philippine *Dilochia* species by its terrestrial habit and is distinguished from all known *Dilochia* species by its monopodial inflorescence, rarely branching in two, and a pale yellow to dull orange or brownish-yellow labellum devoid of purple spots.

## Introduction

*Dilochia* Lindl. is a genus in the Orchidaceae comprising ten species found in Southeast Asia and New Guinea (Thomas 1993; Sulistriarini 2012; Ormerod 2015; Govaerts et al. 2019). Only two species have been previously recorded in the Philippines (Pelser et al. 2011 onwards), *viz. D.elmeri* Ames and *D.wallichii* Lindl., both of which are epiphytic. *D.elmeri* is endemic to the Philippines with extant populations in Luzon (Rizal), Visayas (Samar and Leyte), and Mindanao (Davao), whereas the native range of *D.wallichii* encompasses the Malesian region and Thailand.

The new species was discovered in 2016 by the first author during the botanical survey for the Global Environment Facility (GEF)–funded project, ‘Removing Barriers to Invasive Species Management in Production and Protection Forest in Southeast Asia (FORIS)’, in the Mount Hamiguitan Range Wildlife Sanctuary (MHRWS). Two flowering individuals of *Dilochia* were documented and collected inside a sampling quadrat. Unfortunately, measurements and the description of characters *in situ* were not made before the specimens were pressed and dried. Several flowering individuals were observed and collected during fieldwork with Central Mindanao University (CMU) and the Botanical Research Institute of Texas (BRIT) in June 2019. Furthermore, fruiting specimens were collected in subsequent fieldwork conducted by CMU together with the first author in August 2019.

Photographs and *in situ* descriptive observations of the colors of vegetative and reproductive structures were captured during botanical inventories in 2016 and 2019. Illustrations and further detailed morphological and microscopic examinations of four voucher specimens preserved in denatured alcohol were recorded at the Botany and National Herbarium Division, National Museum of the Philippines. Measurements of important plant parts were made using Mitutoyo Digimatic Caliper. On detailed examination, the authors realized that this species differs greatly from all other species of *Dilochia*. Therefore, we have described and illustrated the new species *Dilochiadeleoniae* Tandang & Galindon, the third species from the Philippines and 11^th^ species in the world.

## Taxonomy

### Dilochia deleoniae

Taxon classificationPlantaeAsparagalesOrchidaceae

Tandang & Galindon sp. nov.

C87E78EB-7299-5E7B-91F3-311471B891D8

urn:lsid:ipni.org:names:77205270-1

Figs 1 , 2

#### Diagnosis.

This species is distinct from the two known epiphytic Philippine species by having an entirely terrestrial habit. Further, among the characters that separate it from other known *Dilochia* species, the new species has a monopodial inflorescence or rarely branching in two. It is similar to *D.beamanii* Ormerod (Ormerod 2015) of Sabah, Malaysia in its reproductive structures. Both species have a terminal flower, with similarity in shape and color of bracts, sepals, and petals. Furthermore, their columns are both winged and with small variation in length. However, the new species has a monopodial inflorescence or rarely branching in two, bearing 7 to 9 flowers (vs. a 3- to 6-branched inflorescence bearing ≥ 13 flowers in *D.beamanii*); dorsal sepal 11-veined, lateral sepal 10-veined, and petal 8-veined (vs. dorsal sepal 5-veined, lateral sepal 5-veined, and petal 7-veined in *D.beamanii*). In addition, the labellum of *D.beamanii* is yellow-ochre with white margins and apex, and with purple spots, whereas the new species has a labellum with a white base and a pale yellow to dull orange or brownish-yellow lip from the disc to the margins and is consistently devoid of purple spots.

**Figure 1.**
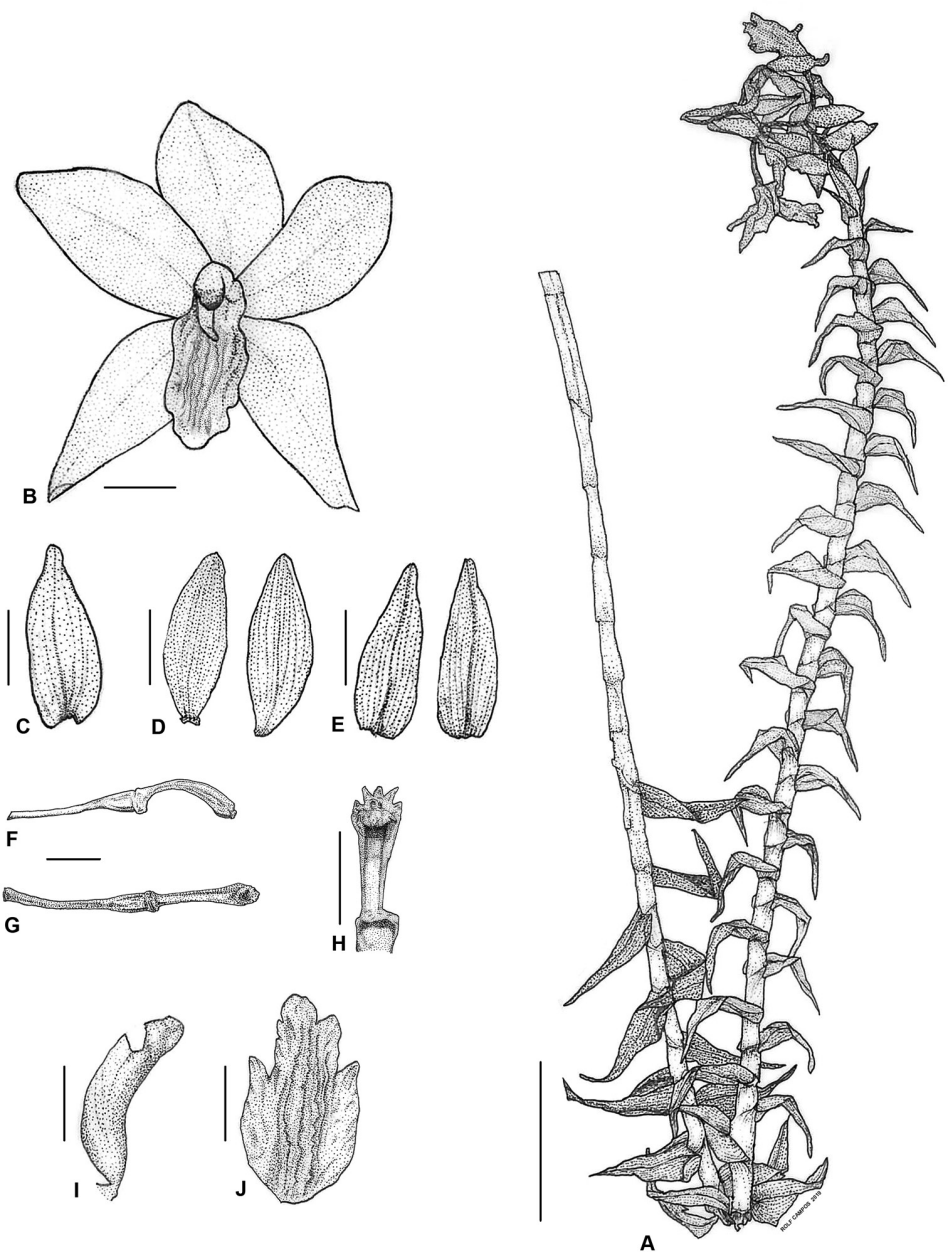
Line drawings of *Dilochiadeleoniae* Tandang & Galindon sp. nov. **A** habit **B** flower **C** dorsal sepal **D** pair of petals **E** pair of lateral sepals **F** side view of the pedicel including the ovary and the column **G** front view of the pedicel including the ovary and the column **H** column **I** side view of the labellum **J** front view of the labellum with flattened side lobes. Scale bars: 5 cm (**A**); 1 cm (**B–J**). Illustrated by Rolf Campos.

#### Type.

Philippines • Mindanao Island. Davao Oriental: San Isidro Municipality, Mount Hamiguitan Range Wildlife Sanctuary, 6°43'47.40"N, 126°10'53.90"E, forest on ultramafic soil, 07 July 2016, E.R. Tadiosa 2059 with D.N. Tandang (***holotype***: PNH; ***isotypes***: CMUH, BRIT, CAHUP).

#### Description.

Terrestrial clump-forming herb, stem 70.0–182.0 cm × 4.0–6.3 mm (with sheaths), terete, erect, leafy throughout becoming leafless near base. ***Leaf sheaths***: purple becoming light green near leaf base, tubular, longer at lower half of stem, 21.4–38.6 mm long, gradually decreasing to last leaf distally. ***Leaves***: light green abaxially, dark green to purplish adaxially, alternate, spreading, curved downward at distal half, glossy, glabrous, lanceolate, usually larger below stem, 57.1–69.6 × 18.7–23.0 mm, smaller ones distally 22.1–23.5 × 9.0–9.7 mm, prominently 7 or 8 parallel veins adaxially; apex attenuate, margin entire, purple to light green. ***Inflorescence***: terminal, racemose, semi-pendulous, to 65.0 mm long, 7- to 9-flowered; *peduncle* light yellow, glabrous, bearing 3 internodes, 23.3–28.4 mm long, enclosed by 3 sterile acuminate peduncular scales; *peduncular scales* cymbiform, parallel veins numerous, vinaceous abaxially, ivory to cream adaxially, apex acute, 16.4–20.4 × 9.8–14.4 mm wide. ***Floral bracts***: clasping pedicels, glabrous, variable in color from yellow green to cream with vinaceous coloration outside at margin and apex, cymbiform, 14.8–21.3 × 6.5–12.4 mm, apex acuminate. ***Pedicel including ovary***: 20.4–23.1 mm long, terete, glabrous, clavate, pale green with occasional purple markings. ***Ovary***: dark purple to green, 6-ribbed. ***Flowers***: with creamy white sepals and petals, fleshy, labellum whitish at the base, becoming pale yellow to dull orange or brownish-yellow except white at apex, underside white with yellow margin at epichilium. ***Dorsal sepal***: cymbiform, lanceolate, 26.9–27.7 × 8.8–9.9 mm, apex obtuse-acute, nerves 11. ***Lateral sepals***: obliquely lanceolate, 26.9–28.8 × 7.2–9.3 mm, apex conduplicate, attenuate, nerves 10. ***Petals***: oblong-oblanceolate, 25.8–26.7 × 8.3–9.5 mm, apex subobtuse, nerves 8. ***Labellum***: oblong-elliptic, 3-lobed, 21.1–21.6 mm long; carinae 5, longitudinal on lip disc, undulating, inner 3 originating from base of labellum to tip, 2 outer shorter than 3 middle, originating from middle part of labellum to middle portion of epichilium; *hypochilium* elliptic, 12.0–12.9 × 9.5–10.0 mm, with erect side lobes, margin entire, apex praemorse, 3-keeled over length of hypochilium, side lobes with numerous parallel veins pointing toward margin; *epichilium* oblong-elliptic, 8.1–8.2 × 4.1–4.9 mm, apex rounded, margin crisped; keels 5, inner 3 continuing from hypochilium, longer than outer 2. ***Column***: slender, 16.2–16.7 × 4.3–5.0 mm, white, light yellow at base, narrow-winged, widened at the apex. ***Fruits***: ovate, 21.9–27.3 mm long, dark purple to green, tinge of purple at ridge, ovate, 6-grooved, perianth persistent. ***Seed***: winged, 1.6–2.0 × 0.1–0.2 mm, white, numerous.

**Figure 2.**
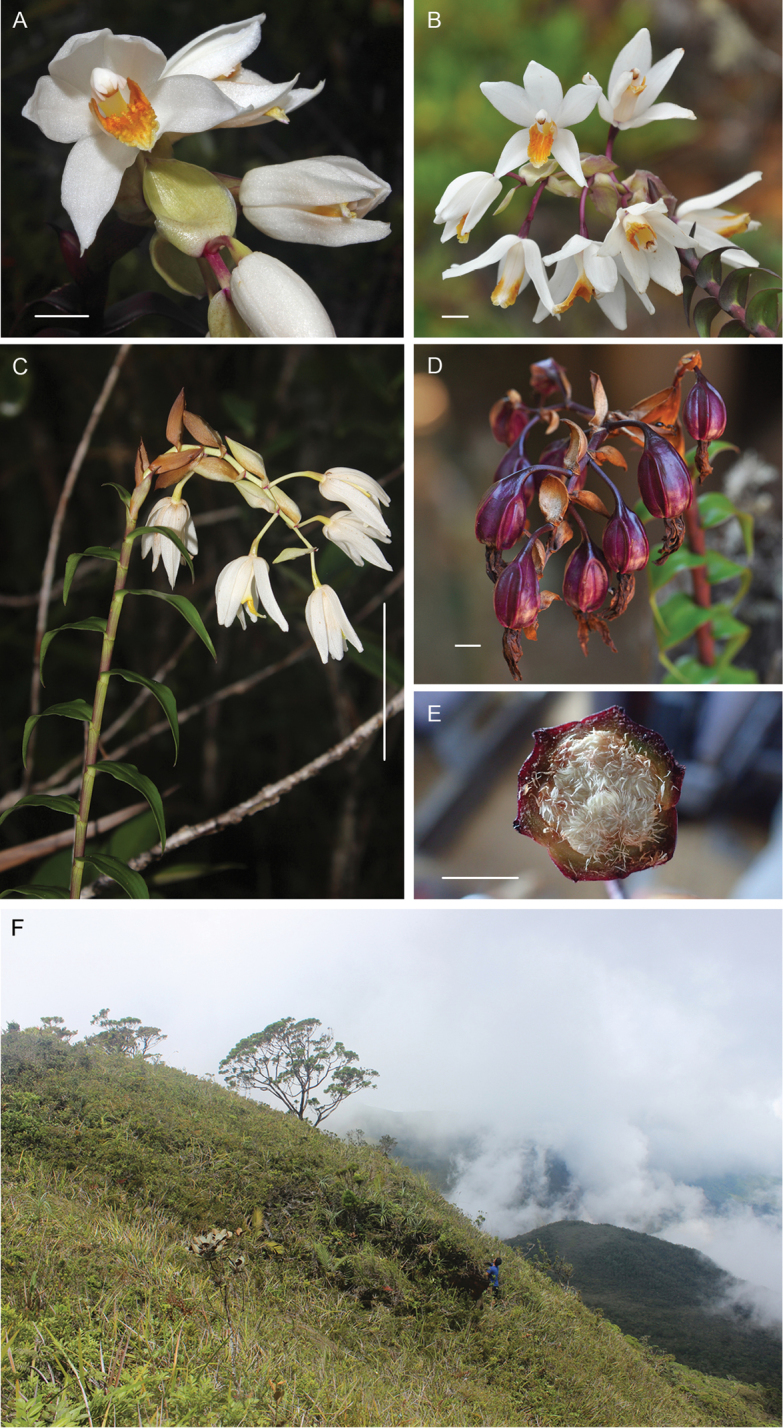
*Dilochiadeleoniae* Tandang & Galindon sp. nov. **A** flower details showing the colorations of the sepals, petals, labellum and bracts **B** racemose inflorescence **C** semi-pendulous inflorescence and the alternate arrangement of the leaves **D** dark-purpled fruits **E** cross section of the fruit showing numerous whitish seeds **F** habitat on the summit of Mount Hamiguitan, forest over ultramafic soils. Scale bars: 1 cm (**A, B, D, E**); 5 cm (**C**). **A, C, F** photos by John Michael M. Galindon **B, D, E** photos by Danilo N. Tandang.

#### Distribution.

Endemic to the Philippines. Mindanao Island, Davao Oriental, San Isidro Municipality, Mount Hamiguitan Range Wildlife Sanctuary.

#### Habitat and ecology.

This terrestrial orchid species prefers open to partly shaded habitat, where it is found in clumps or scattered. The populations rarely occur in forest over ultramafic rocks between elevations ca. 1100–1200 meters above sea level (m a.s.l.) but is common in the pygmy forest ecosystem at the mountain summit between 1560 and 1650 m a.s.l. This new species was recorded inside a sampling quadrat, growing with other native orchid species such as *Appendiculatembuyukenensis* J.J.Wood and *Dendrochilumkopfii* Lückel., and with other species such as *Agathis* sp., *Dacrydiumbeccarii* Parl., *Dacrydiumelatum* (Roxb.) Wall. ex Hook., *Falcatifoliumgruezoi* de Laub., *Gleicheniavulcanica* Blume, *Leptospermumjavanicum* Blume, *Machaerinadisticha* (C.B.Clarke) T.Koyama, *M.glomerata* (Gaudich.) T.Koyama, *Medinillamyrtiformis* (Naudin) Triana, *M.theresae* Fernando, *Myrsineamorosoana* Pipoly, *Scaevolamicrantha* C.Presl, *Symplocospolyandra* (Blanco) Brand, *Tasmanniapiperita* (Hook.f.) Miers, and *Vaccinium* spp.

#### Additional specimens examined.

Philippines • Mindanao Island, Davao Oriental Province, Municipality of San Isidro, Mount Hamiguitan Range Wildlife Sanctuary; 6°43'49.26"N, 126°10'48.22"E; 1204 m elevation; 18 June 2019; Plants and Lichens of the Southern Philippines Survey 758 (BRIT, CMUH, PNH) • Mindanao Island, Davao Oriental Province, Municipality of San Isidro, Mount Hamiguitan Range Wildlife Sanctuary; 6°43'49.15"N, 126°10'45.41"E, 1184 m elevation, 18 June 2019, Plants and Lichens of the Southern Philippines Survey 1316 (BRIT, CMUH, PNH).

#### Etymology.

The new species is named after Ms Josefina De Leon, the former Chief of the Wildlife Resources Division under the Biodiversity Management Bureau of the Department of Environment and Natural Resources, who has pursued wildlife conservation for more than 35 years and who remains a biodiversity conservation advocate. During her time in the Bureau, the FORIS project was launched and researchers from the National Museum of the Philippines were invited to be part of the technical working group that led to the discovery of the new species.

#### Conservation status.

*Dilochiadeleoniae* is only known from the 68.34 km^2^ Mount Hamiguitan Range Wildlife Sanctuary and is confined to its high elevations particularly in mossy-pygmy forests. The extent of occurrence is < 100 km^2^ and area of occupancy is 8 km^2^, as based on *GeoCAT* (Bachman et al. 2011; http://geocat.kew.org/) with the default 2 km^2^ grid. The mountain range was declared a protected area under Republic Act No. 9303, and was recently designated as a UNESCO World Heritage Site, helping to protect this species from habitat degradation, poaching, and over-collection. However due to ‘restricted area of occupancy’ and the possible effect of continuous poaching and climate change, this species is ‘capable of becoming critically endangered or extinct within a very short time’. Therefore, following the IUCN Categories and Criteria (IUCN 2012), we classify this species as Vulnerable [VU D2].

## Supplementary Material

XML Treatment for Dilochia deleoniae
